# Respiratory Heterogeneity Shapes Biofilm Formation and Host Colonization in Uropathogenic Escherichia coli

**DOI:** 10.1128/mBio.02400-18

**Published:** 2019-04-02

**Authors:** Connor J. Beebout, Allison R. Eberly, Sabrina H. Werby, Seth A. Reasoner, John R. Brannon, Shuvro De, Madison J. Fitzgerald, Marissa M. Huggins, Douglass B. Clayton, Lynette Cegelski, Maria Hadjifrangiskou

**Affiliations:** a Department of Pathology, Microbiology, and Immunology, Vanderbilt University Medical Center, Nashville, Tennessee, USA; b Department of Chemistry, Stanford University, Stanford, California, USA; c Division of Pediatric Urology, Vanderbilt University Medical Center, Nashville, Tennessee, USA; d Vanderbilt University, Nashville, Tennessee, USA; e Vanderbilt Institute for Infection, Immunology and Inflammation, Vanderbilt University Medical Center, Nashville, Tennessee, USA; Columbia University; California Institute of Technology

**Keywords:** *Escherichia coli*, biofilms, heterogeneity, oxygen gradients, respiration, urinary tract infection

## Abstract

Biofilms are multicellular bacterial communities encased in a self-secreted extracellular matrix comprised of polysaccharides, proteinaceous fibers, and DNA. Organization of these components lends spatial organization to the biofilm community such that biofilm residents can benefit from the production of common goods while being protected from exogenous insults. Spatial organization is driven by the presence of chemical gradients, such as oxygen. Here we show that two quinol oxidases found in Escherichia coli and other bacteria organize along the biofilm oxygen gradient and that this spatially coordinated expression controls architectural integrity. Cytochrome *bd*, a high-affinity quinol oxidase required for aerobic respiration under hypoxic conditions, is the most abundantly expressed respiratory complex in the biofilm community. Depletion of the cytochrome *bd-*expressing subpopulation compromises biofilm complexity by reducing the abundance of secreted extracellular matrix as well as increasing cellular sensitivity to exogenous stresses. Interrogation of the distribution of quinol oxidases in the planktonic state revealed that ∼15% of the population expresses cytochrome *bd* at atmospheric oxygen concentration, and this population dominates during acute urinary tract infection. These data point toward a bet-hedging mechanism in which heterogeneous expression of respiratory complexes ensures respiratory plasticity of E. coli across diverse host niches.

## Abstract (precis)

Biofilms are multicellular bacterial communities encased in a self-secreted extracellular matrix comprised of polysaccharides, proteinaceous fibers, and DNA. Organization of these components lends spatial organization in the biofilm community. Here we demonstrate that oxygen gradients in uropathogenic Escherichia coli (UPEC) biofilms lead to spatially distinct expression programs for quinol oxidases—components of the terminal electron transport chain. Our studies reveal that the cytochrome *bd*-expressing subpopulation is critical for biofilm development and matrix production. In addition, we show that quinol oxidases are heterogeneously expressed in planktonic populations and that this respiratory heterogeneity provides a fitness advantage during infection. These studies define the contributions of quinol oxidases to biofilm physiology and suggest the presence of respiratory bet-hedging behavior in UPEC.

## INTRODUCTION

Rather than existing as phenotypically uniform populations, bacterial colonies, biofilms, and cultures are characterized by subpopulations that are often phenotypically distinct. This intrastrain heterogeneity can be irreversible, through the acquisition of mutations, or transient and reversible if it is brought about by stochastic differences in the abundance and activity of regulators in each individual cell or by metabolic adaptation to local environmental conditions. Heterogeneity often confers a survival advantage to the population by allowing at least portion of the population to survive in a given niche.

In biofilms, bacteria assemble in an organized fashion in three-dimensional space. One of the most critical features of biofilms is a self-secreted extracellular matrix (ECM) that comprises a variety of exopolysaccharides, proteinaceous fibers, and extracellular DNA (1). The ECM protects the biofilm residents from predation, desiccation, assault by antimicrobial agents, and—when biofilms form in the host—the immune system. In addition to providing a physical barrier against external threats, the ECM also serves as a barrier to diffusion. Restricted diffusion, in conjunction with the metabolic activity of resident bacteria, leads to the establishment of various chemical gradients throughout the biofilm community (2, 3). Bacteria at different locales along the gradient respond to the microenvironment differently and as a result differentiate into distinct and often metabolically cooperative subpopulations (2–6). Previous studies in Pseudomonas aeruginosa and Escherichia coli indicated that oxygen gradients play a key role in regulating the differential expression of genes involved in biofilm formation and metabolic specialization (7–10). The presence of an oxygen gradient suggests the emergence of subpopulations that utilize different respiratory components as a function of the oxygen abundance to which they are exposed. This leads to the hypothesis that the metabolic programs of differentially respiring subpopulations are distinct from one another and contribute to differential production of biofilm goods that in turn enhance biofilm resilience.

Escherichia coli is a facultative anaerobe capable of utilizing multiple metabolic pathways to fulfill its energy requirements. In aerobically respiring E. coli, quinol oxidases comprise essential components of the terminal electron transport chain that couple the flow of electrons to the reduction of molecular oxygen into water (11, 12). E. coli encodes two classes of quinol oxidases with differing oxygen affinities: one heme copper oxidase, cytochrome *bo* (encoded by the *cyoABCD* gene cluster), and two *bd-*type oxidases, cytochromes *bd* (*cydABX*) and *bd*_2_ (*appBC*) (12, 13). Studies in K-12 E. coli indicated that cytochrome *bo* is induced at high (atmospheric, 21%) oxygen tensions, whereas the *bd*-type oxidases are induced at low (hypoxic, 2 to 15%) oxygen tensions (12, 14). Cytochromes *bd* and *bd*_2_ have approximately 60% amino acid identity, similar spectral properties, and indistinguishable reaction mechanisms (13, 15). Based on the *in vitro* expression patterns of these quinol oxidases, we hypothesized that cytochrome *bo* would be enriched on the air-exposed biofilm surface, whereas cytochromes *bd* and *bd*_2_ would be enriched in the hypoxic interior.

Here we report that the spatial distribution of quinol oxidases in biofilms formed by uropathogenic Escherichia coli (UPEC) is a fundamental driver of biofilm architecture. Peptide nucleic acid fluorescence *in situ* hybridization (PNA-FISH) analyses assigned locations to each quinol oxidase-producing subpopulation, elucidating for the first time spatially distinct expression programs for respiratory oxidases in E. coli. Depletion of the cytochrome *bd*-expressing subpopulation from the biofilm significantly impaired diffusion resistance by altering the abundance and organization of the ECM. Assessment of deletion mutants in a well-established urinary tract infection murine model revealed that only the cytochrome *bd* mutant was significantly attenuated for virulence, although the infecting pool of bacteria in the parent strain exhibited heterogeneous expression of all three respiratory oxidases. *In situ* analysis of urine-associated bacteria demonstrated a shift of the population to cytochrome *bd* expression, suggesting that the bladder favors cytochrome *bd*-expressing bacteria and that heterogeneity in the input pool provides a fitness advantage to uropathogenic strains. Our studies, performed on one of the most commonly acquired human pathogens and a prolific biofilm producer *in vivo*, unveil a potential avenue for targeting heterogeneity and homogenizing bacterial programming as a therapeutic approach.

## RESULTS

### Cytochrome *bd* is the most abundant respiratory transcript in mature UPEC biofilms.

**FIG 1:**
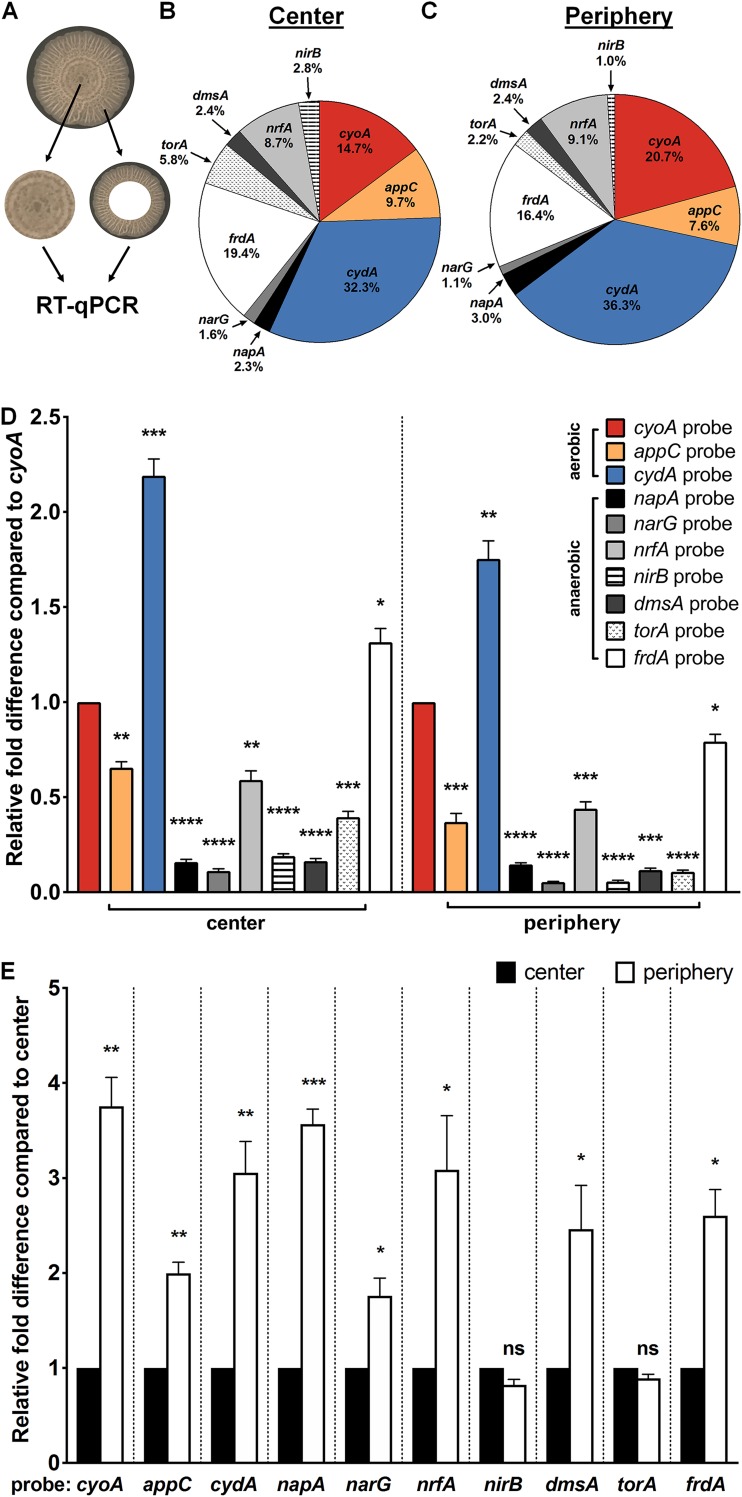
Lateral expression of respiratory complexes in Escherichia coli biofilms. (A) Image of a mature colony biofilm formed by UPEC strain UTI89 on YESCA agar without supplementation of alternative terminal electron acceptors. The center and periphery of colony biofilms, including both the surface and interior of each region, were harvested and subjected to RNA extraction and RT-qPCR using probes targeting each respiratory operon present in UPEC. (B and C) Pie charts indicating the relative abundance of detected respiratory transcripts in the biofilm center (B) and periphery (C). Aerobic respiratory operons are presented in color, whereas anaerobic respiratory operons are presented in grayscale. (D) Graph depicting relative fold differences in respiratory transcript abundance in the biofilm center and periphery compared to *cyoA* abundance in the same region. (E) Graph depicting relative fold difference in abundance of each transcript in the biofilm periphery compared to abundance of the same transcript in the biofilm center. The graphs and pie charts depict the average from four biological replicates. Statistical analysis was performed in GraphPad Prism using a two-tailed paired *t* test. Data are presented as mean ± SEM. *, *P* < 0.05; **, *P* < 0.01; ***, *P* < 0.001; ****, *P* < 0.0001.

In previous studies we reported spatial organization of proteins as a function of oxygen gradients in surface-associated UPEC biofilms formed at the air-liquid interface of yeast extract-Casamino Acids medium (YESCA) (9), which induces expression of key matrix components (curli amyloid fibers, type 1 pili, secreted proteins Hu-α/β, and cellulose) that are also critical for fitness in the urinary tract (9, 16–18). We then went on to show that biofilm formation is greatly diminished under anaerobic conditions, despite the addition of alternative terminal electron acceptors used by E. coli for anaerobic respiration and irrespective of growth medium (19). Given that aerobic respiration is critical for UPEC colonization and the establishment of intracellular biofilms during bladder infection (20–22), we sought to determine the relative expression of aerobic and anaerobic respiratory machineries in mature colony biofilms formed under aerobic conditions on YESCA agar without supplementation of alternative terminal electron acceptors. Under these growth conditions, UPEC forms elaborate rugose-colony biofilms (Fig. 1A) that quickly establish an oxygen gradient from the surface to the interior of the biofilm (8). Previous studies demonstrated the presence of a matrix-rich region and a matrix-devoid region in colony biofilms imaged at 48 h and 6 days post-inoculation (8, 23–25) and additionally revealed distinct spatial expression of regulators at the growing edge versus the center of the biofilm (23). To investigate the role of quinol oxidases in biofilms, we first used RT-qPCR to measure the steady-state transcript at the growing edge (periphery) and the center of the colony (Fig. 1; see also Fig. S1 in the supplemental material). Although overall transcript abundance was significantly increased in the periphery relative to the center (Fig. 1E), consistent with the notion that cells at the periphery are more metabolically active, we observed a similar distribution of transcript at the center of the biofilm and the growing edge (Fig. 1B to D). Consistent with previous studies demonstrating the importance of aerobic respiration in UPEC biofilms, the majority of detected transcript corresponded to aerobic respiratory components (Fig. 1B and C). The most abundant transcript was that of *cydA* (Fig. 1B to D and Fig. S1), corresponding to cytochrome *bd* complex (*cydABX*) expression. The *cydA* transcript levels were approximately 2-fold higher than those corresponding to *cyoABCD* (Fig. 1B to D), which was the second most highly abundant oxidase under the conditions tested. Although most anaerobic respiratory operons exhibited baseline expression levels, we detected high levels of transcript corresponding to fumarate reductase (*frdA*) and periplasmic nitrite reductase (*nrfA*) (Fig. 1B to D and Fig. S1). These results reveal the presence of marked respiratory heterogeneity within UPEC biofilm communities and suggest that respiration via cytochrome *bd* may be preferred.

10.1128/mBio.02400-18.1 FIG S1 Expression of respiratory complexes compared to *gyrB*. (A) Graph depicting relative abundance of each respiratory transcript in the center and periphery of day 11 colony biofilms compared to *gyrB*. (B) Average *C_T_* values for each transcript in the center and periphery of colony biofilms. Data are presented as mean ± SEM. All data are representative of four biological replicates. Download FIG S1, TIF file, 0.9 MB. Copyright © 2019 Beebout et al. 2019 Beebout et al. This content is distributed under the terms of the Creative Commons Attribution 4.0 International license.

### Spatial organization of quinol oxidase expression along the oxygen gradient.

**FIG 2:**
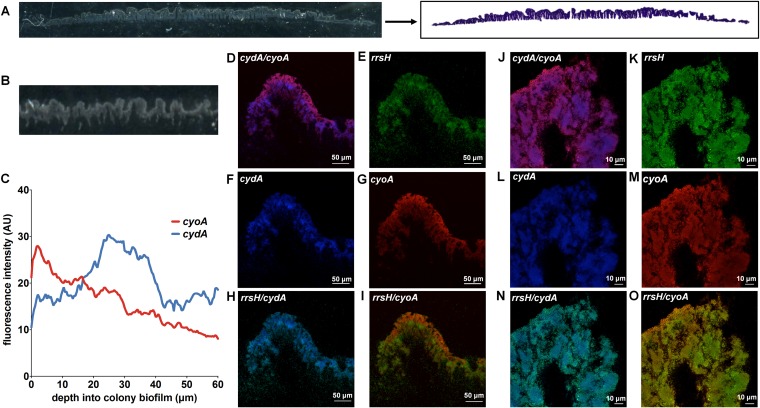
Expression of quinol oxidases as a function of the oxygen gradient. (A) Representative images depicting a biofilm cryosection before (left) and after (right) fixation and crystal violet staining. (B) Magnified image of a cryosection allows visualization of architectural features of the biofilm. (C) Fluorescence intensity of *cyoA* and *cydA* PNA-FISH probes was quantified on ImageJ. Data are presented as the average fluorescence intensity as a function of depth obtained from four images, each with five measurements per image. (D to O) Representative images of PNA-FISH-stained biofilm cryosections at ×20 magnification (D to I) and ×63 magnification (J to O). Cryosections were stained with PNA-FISH probes targeting *cyoA* and *cydA* with *rrsH* (16S rRNA) as an endogenous control. Images are representative of three biological replicates.

To define the spatial distribution of quinol oxidase-expressing subpopulations, we performed PNA-FISH on biofilm cryosections of mature colony biofilms using probes targeting each quinol oxidase operon (*cyoA*, *appC*, and *cydA*) as well as *rrsH* as an endogenous control (Fig. 2). Because cryosectioning captures both macroscopic and microscopic architecture of biofilms with minimal disruption to the overall structure or organization of the resident bacteria, this approach allows us to define the *in situ* distribution of transcripts in unperturbed communities (Fig. 2A and B). Each PNA-FISH probe was designed using the validated probe sequences used for qPCR (Table S1) to ensure comparable hybridization efficiencies for each probe. Specificity of each probe was confirmed using RT-qPCR (see Fig. 4H) and through staining of planktonic cells (Fig. S2). SYTO 9 staining of sections was used as an additional control to localize the entire biofilm community and account for possible hybridization inconsistencies with the *rrsH* control probe (Fig. 2E and K and Fig. S3). To account for possible mislocalization of signal due to biofilm breakage during the cryosectioning and staining procedure, we focused our analysis on regions devoid of significant breaks in the cryosection. Consistent with previous observations demonstrating that the highest oxygen abundance is at the air-exposed surface of the biomass (7, 8), we observed that *cyoABCD* transcript was most abundant in bacteria lining air-exposed surfaces of the biofilm (Fig. 2C, D, G, I, J, M, and O). In contrast, the highest abundance of *cydABX* transcript was found in densely packed clusters of bacteria in the interior of the biofilm (Fig. 2C, D, F, H, J, L, and N). Although cytochromes *bd* and *bd*_2_ are both induced under oxygen-limited conditions (13, 26), we observe different transcript distribution for these two gene clusters. Rather than organizing along the oxygen gradient, *appBC* transcript was observed to be evenly distributed throughout the community (Fig. S3). Interestingly, we also observe basal expression of cytochrome *bo* across the community with enrichment of cytochrome *bd* in pockets of cells in the interior (Fig. 2J to O), suggesting that individual cells may express multiple quinol oxidases simultaneously within biofilms. Additionally, while many biofilm wrinkles are empty or sparsely populated with *rrsH-*staining cells, we observe other wrinkles that are densely populated (Fig. S4). We observe reduced intensity of quinol oxidase staining in the interior of those populated wrinkles (Fig. S4), suggesting that—consistent with previous reports (7, 27)—respiration in the deeper layers of the biofilm respiration occurs anaerobically. Based on the qPCR results (Fig. 1B to D), respiration in the populated wrinkles may be occurring via fumarate reductase or periplasmic nitrite reductase. Here we focused our studies on understanding the contribution of cytochrome *bd* to biofilm architecture.

10.1128/mBio.02400-18.2 FIG S2 PNA-FISH on quinol oxidase deletion mutants. Representative images of Δ*cydAB* (A to D) and Δ*cyoAB* (E to H) cells stained with PNA-FISH probes to assess specificity. Download FIG S2, TIF file, 2.0 MB. Copyright © 2019 Beebout et al. 2019 Beebout et al. This content is distributed under the terms of the Creative Commons Attribution 4.0 International license.

10.1128/mBio.02400-18.3 FIG S3 Localization of *appC* transcript in biofilm cryosections. (A) Representative image of SYTO 9-stained biofilm cryosection at ×20 magnification. (B and C) Representative images of biofilm cryosections stained with an *appC* PNA-FISH probe at ×20 (B) and ×63 (C) magnification. Download FIG S3, JPG file, 0.4 MB. Copyright © 2019 Beebout et al. 2019 Beebout et al. This content is distributed under the terms of the Creative Commons Attribution 4.0 International license.

10.1128/mBio.02400-18.4 FIG S4 Localization of quinol oxidase transcripts in biofilm wrinkles. Representative image of a PNA-FISH-stained cryosection depicting a biofilm wrinkle with a central region filled with *rrsH-*stained cells at ×20 magnification. Download FIG S4, JPG file, 2.4 MB. Copyright © 2019 Beebout et al. 2019 Beebout et al. This content is distributed under the terms of the Creative Commons Attribution 4.0 International license.

10.1128/mBio.02400-18.10 TABLE S1 Primers and probes. Download Table S1, DOCX file, 0.1 MB. Copyright © 2019 Beebout et al. 2019 Beebout et al. This content is distributed under the terms of the Creative Commons Attribution 4.0 International license.

### Loss of cytochrome *bd* alters biofilm architecture, development, and ECM abundance.

**FIG 3:**
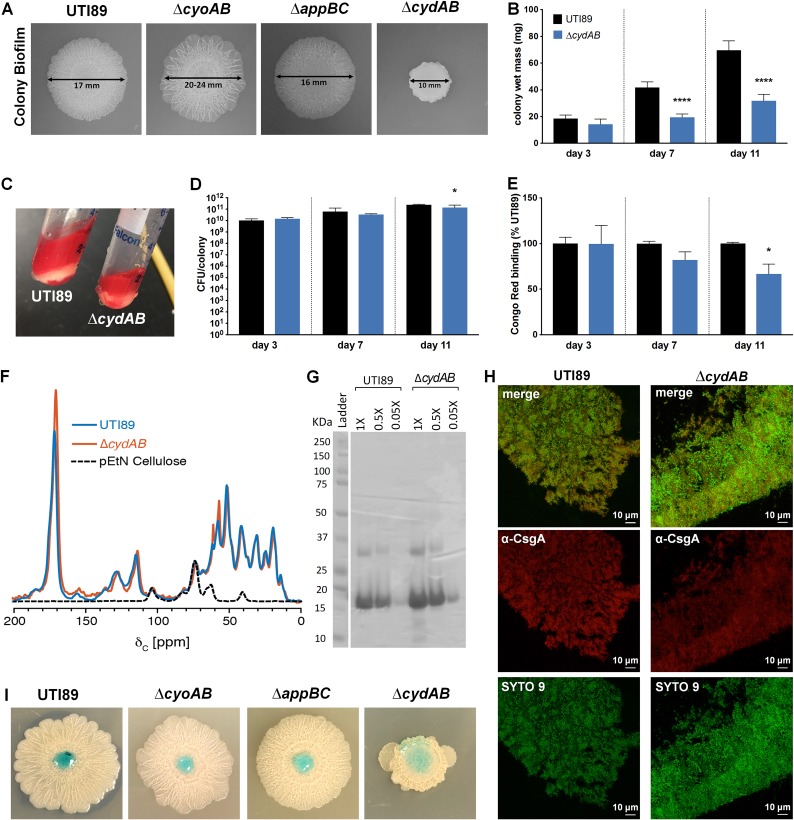
Cytochrome *bd* organizes biofilm architecture and ECM production. (A) Colony biofilms of UTI89 and quinol oxidase mutants grown on YESCA agar for 11 days. Images are representative of at least 30 biological replicates. (B) Graph depicting wet mass of individual colony biofilms at days 3, 7, and 11 of growth. Data are the average from five biological replicates per day. Data are presented as mean ± SD. (C) Image depicting gross changes to extracellular matrix (ECM) abundance between UTI89 and Δ*cydAB* colony biofilms. ECM is stained red by the presence of Congo red in the growth medium. (D) CFU per colony biofilm was measured at days 3, 7, and 11 of growth. Data are presented as mean ± SD. Data are representative of five biological replicates. (E) Congo red binding as a percentage of binding in UTI89. Data are presented as mean ± SEM. (F) Solid-state NMR spectra of the ECM of UTI89 (blue), Δ*cydAB* strain (orange), and isolated pEtN cellulose (black). (G) SDS-PAGE gel of UTI89 and Δ*cydAB* ECM. ECM was treated with 98% formic acid and vacuum centrifuged prior to analysis to dissociate curli amyloid fibers. (H) Immunofluorescence images of curli (α-CsgA, red) localization in UTI89 and Δ*cydAB* colony biofilm cryosections. (I) Colored water droplets were added to the top of day 11 colony biofilms to probe biofilm barrier function. All statistical analysis was performed in GraphPad Prism using a two-tailed unpaired *t* test. *, *P* < 0.05; **, *P* < 0.01; ***, *P* < 0.001; ****, *P* < 0.0001.

The highly ordered spatial organization of cytochrome *bo* and cytochrome *bd* in the biofilm raised the hypothesis that each of these quinol oxidase-expressing subpopulations uniquely contributes to overall biofilm architecture. To test this hypothesis, we created isogenic deletion mutants lacking the *cyoAB*, *appBC*, or *cydAB* genes and compared the biofilms formed by the resulting strains (Fig. 3A). Colony biofilms formed by the parental strain expand to an average diameter of 16.8 mm over an 11-day incubation period and exhibit elaborate rugose architecture with distinct central and peripheral regions (Fig. 3A and Fig. S5). Strains deleted for *cyoAB* and *appBC* exhibited inverse phenotypes to each other, with the Δ*cyoAB* colony biofilms expanding more than the parental strain (average diameter, 19.9 mm) and the Δ*appBC* colony biofilms appearing more compact and with apparently higher rugosity (Fig. 3A and Fig. S5). Strikingly, while Δ*cyoAB* and Δ*appBC* colony biofilms displayed only minor architectural changes, Δ*cydAB* colony biofilms exhibited pronounced defects in both development and architecture (Fig. 3A and Fig. S5). Colony biofilms from all strains grew at similar rates for the first 72 h (Fig. S5). However, Δ*cydAB* colony growth was significantly stunted between days 3 and 11, with radial expansion remaining at an average diameter of 10.3 mm and colonies exhibiting a wet mass approximately 50% of the parental strain after 7 days of growth, even though the CFU produced by the two strains were comparable (Fig. 3B and D and Fig. S5). Complementation of the Δ*cydAB* strain with an extrachromosomal construct expressing *cydABX* under its native promoter rescued the deletion phenotype, indicating that the defects observed in the Δ*cydAB* mutant stem solely from the removal of the *cydABX* cluster (Fig. S7). Furthermore, deletion of both *cyoAB* and *appBC* from the same strain led to an early onset of rugose phenotype (Fig. S6). Together, these results demonstrate that cytochrome *bd* is a key contributor to biofilm development and suggest that loss of *cydAB* alters the synthesis and organization of the ECM.

10.1128/mBio.02400-18.5 FIG S5 Temporal development of colony biofilms by UTI89 and quinol oxidase mutants. (A) Representative images of UTI89 and quinol oxidase mutant colony biofilms grown on YESCA agar taken on days 3, 7, and 11 of growth. (B) Graph depicting colony biofilm diameter at days 3, 7, and 11 of growth. Each triangle represents an individual colony biofilm. Data are representative of at least 30 biological replicates. Statistical analysis was performed in GraphPad Prism using Welch’s *t* test. *, *P* < 0.05; **, *P* < 0.01; ***, *P* < 0.001; ****, *P* < 0.0001. Download FIG S5, TIF file, 2.6 MB. Copyright © 2019 Beebout et al. 2019 Beebout et al. This content is distributed under the terms of the Creative Commons Attribution 4.0 International license.

10.1128/mBio.02400-18.6 FIG S6 Analysis of *ΔappBC ΔcyoAB*::*kanR* colony biofilms. Comparison of colony biofilms formed by UTI89, *ΔcyoAB*, *ΔappBC*, *ΔappBC ΔcyoAB*::*kanR*, and *ΔcydAB* strains after 6 days of growth on YESCA agar. Images are representative of five biological replicates. Download FIG S6, TIF file, 2.3 MB. Copyright © 2019 Beebout et al. 2019 Beebout et al. This content is distributed under the terms of the Creative Commons Attribution 4.0 International license.

10.1128/mBio.02400-18.7 FIG S7 Extrachromosomal complementation of Δ*cydAB* rescues biofilm defects. Representative images on UTI89_pTRC99a, *ΔcydAB*_pTRC99a, and complemented *ΔcydAB*_pCydABX strains under the control of a native promoter. Images were taken of colony biofilms grown on YESCA agar at days 3, 7, and 11 of growth. Images are representative of at least five biological replicates. Download FIG S7, JPG file, 0.2 MB. Copyright © 2019 Beebout et al. 2019 Beebout et al. This content is distributed under the terms of the Creative Commons Attribution 4.0 International license.

Under the conditions used, the ECM of E. coli comprises primarily cellulose and curli amyloid fibers (28). Previous solid-state nuclear magnetic resonance (NMR) spectroscopy analyses on intact ECM material defined the contributions of cellulose and curli to the E. coli biofilm ECM and determined that curli and cellulose are present in a 6-to-1 ratio (28). More recently, the ECM cellulose was determined to be a chemically modified form of cellulose, specifically phosphoethanolamine (pEtN) cellulose (29). To interrogate the effects of *cydAB* deletion on curli and exopolysaccharide production, we extracted ECM and performed solid-state NMR analysis to evaluate the abundance of curli and cellulose components (Fig. 3F and G). The NMR spectra obtained for the parent and Δ*cydAB* ECM are very similar overall, indicating a comparable protein-to-polysaccharide ratio between the samples (Fig. 3F). Consistent with this analysis, we observe no change in protein composition between the parent and Δ*cydAB* ECM samples when analyzed on SDS-PAGE gels (Fig. 3G). We additionally do not observe any overt alterations to curli abundance or localization between UTI89 and Δ*cydAB* biofilm cryosections using immunofluorescence (Fig. 3H). Despite the similar composition, the total amount of ECM recovered was reduced in the Δ*cydAB* biofilm, indicative of a decrease in ECM production. When quantified by Congo red depletion assays, Δ*cydAB* colony biofilms exhibited a trend toward reduced total ECM abundance at 7 days (82.1% of parental value) and significantly reduced abundance at 11 days (66.6% of parental value) (Fig. 3E), which could be the result of reduced CFU at the 11-day time point. Because the protein-to-polysaccharide ratio and curli abundance are unchanged between the parent and Δ*cydAB* biofilms, these data are suggestive of a change to the overall mixture of matrix components in Δ*cydAB* biofilm, with particular reductions in the abundance of non-curli and non-pEtN cellulose ECM components.

The ECM plays a central role in biofilm physiology by providing physical protection against exogenous insults, serving as a structural scaffold, and helping to establish chemical gradients which lead to metabolic differentiation and subpopulation formation (2, 3, 16, 30). As such, disruptions to the matrix can have catastrophic consequences for the biofilm community. We hypothesized that the altered matrix abundance and architecture in the Δ*cydAB* mutant would render the biofilm more susceptible to exogenous insults. To investigate this possibility, we probed the barrier function of the quinol oxidase mutant biofilms by applying a drop of colored water to the surface of mature colony biofilms (Fig. 3I) (31). While the parent strain, Δ*cyoAB*, and Δ*appBC* biofilms repelled the drop, the solution readily penetrated Δ*cydAB* biofilms, demonstrating that the alterations to Δ*cydAB* biofilm architecture and matrix abundance increase penetrance of aqueous solutions.

### Loss of cytochrome *bd* increases population sensitivity to nitrosative stress under ambient oxygen concentrations.

Together, our studies indicate that cytochrome *bd* is highly expressed in biofilms and that loss of the cytochrome *bd*-expressing subpopulation impairs barrier function and reduces the abundance of extracellular matrix. These data suggest that the cytochrome *bd*-expressing subpopulation plays a critical role in promoting ECM synthesis and providing structural integrity to the community. However, it is also possible that cytochrome *bd* is preferentially expressed in the biofilm because cytochrome *bd* provides protection against oxidative and nitrosative stress—by-products of biofilm metabolism (32, 33) and, in the case of infection, components of the innate immune response (34–36). In addition to functioning as a respiratory quinol:O_2_ oxidoreductase, previous studies demonstrated that cytochrome *bd* has catalase activity, is capable of oxidizing the respiratory inhibitor nitric oxide, and is insensitive to nitrosative stress due to its unusually high nitric oxide dissociation rate (34). These biochemical activities are thought to occur at unique locations on the protein; quinol oxidation occurs at the periplasmic Q loop, oxygen reduction and nitric oxide binding occur at heme *d*, and catalase activity is thought to occur through heme *b*_595_ (13, 37, 38). In contrast, cytochrome *bo* affords no protection against nitrosative stress and is irreversibly inhibited by nitric oxide (34).

**FIG 4:**
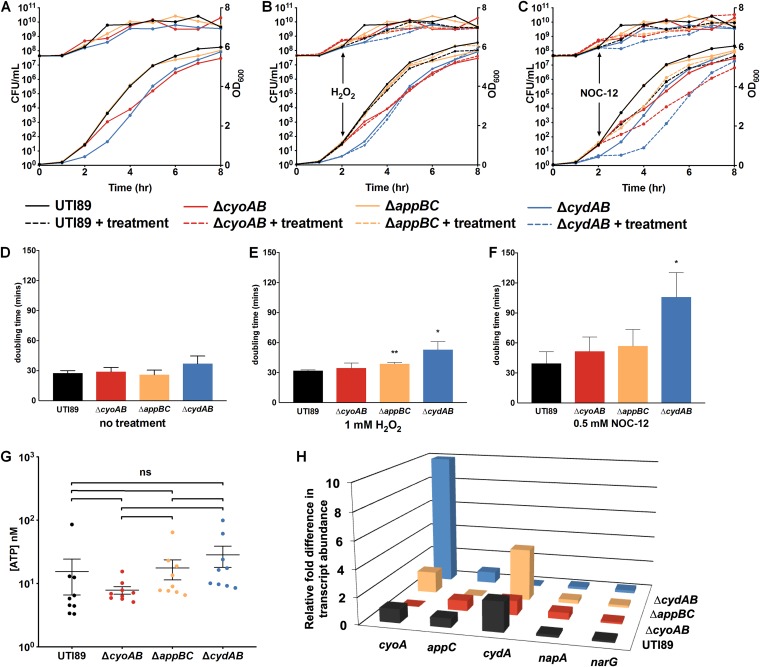
Cytochrome *bd* provides nitrosative stress resistance. (A to C) Growth curves for UTI89, Δ*cyoAB*, Δ*appBC*, and Δ*cydAB* strains as measured by CFU-per-ml (upper lines, left axis) and OD_600_ (lower lines, right axis) with no treatment (A), treatment with 1 mM hydrogen peroxide (B), or treatment with 0.5 mM nitric oxide donor NOC-12 (C). (D to F) Doubling time in minutes of each strain between hours 2 and 4 was calculated using CFU-per-ml data shown in panels A to C. (D) No treatment. (E) Treatment with 1 mM hydrogen peroxide. (F) Treatment with 0.5 mM NOC-12. (G) ATP levels measured from logarithmic cultures of each strain normalized to OD_600_ = 0.5. (H) RT-qPCR data depicting relative fold difference in respiratory transcript abundance in the center of day 11 colony biofilms in UTI89 and quinol oxidase mutant strains. In UTI89 (black), data are presented as relative fold difference in abundance of each transcript compared to *cyoA* abundance. In each mutant strain, data are presented as relative fold difference in transcript abundance compared to the abundance of the same transcript in UTI89. Statistical analysis was performed on GraphPad Prism using a two-tailed unpaired *t* test. All data are presented as mean ± SEM and are representative of at least three biological replicates.

Given these additional functions of cytochrome *bd*, we performed growth curves at ambient oxygen concentration and evaluated the effects of nitrosative and oxidative stress on the fitness of cells lacking each quinol oxidase compared to the parental strain. Without the addition of stressors, both Δ*cydAB* and Δ*cyoAB* mutants exhibited a delay in growth, but growth of the Δ*appBC* strain closely mirrored the parental strain (Fig. 4A and D). Despite the delay, all strains reached similar maximal CFU/ml by the end of the experiment (Fig. 4A). ATP measurements of normalized samples taken from each strain during logarithmic phase revealed no significant overall differences in ATP concentrations (Fig. 4G). Next, to determine whether loss of cytochrome *bd* impairs resistance to oxidative and nitrosative stress, we measured growth with and without these stressors. Consistent with the reported catalase activity of cytochrome *bd*, significant increases in the doubling time of both Δ*cydAB* and Δ*appBC* strains were observed after treatment with 1 mM H_2_O_2_ (Fig. 4B and E). Although previous studies in K-12 E. coli demonstrated that treatment with 1 mM H_2_O_2_ reduced the growth rate of the Δ*cyoAB* strain by ∼70% relative to wild type (39), we did not observe significant reductions in growth rate of the Δ*cyoAB* strain after treatment (Fig. 4B and E). Addition of the nitric oxide donor NOC-12 to planktonic cultures induced an apparent growth delay in all strains but only significantly reduced the growth rate of the Δ*cydAB* mutant (Fig. 4C and F). Whereas treatment with NOC-12 increased the doubling time from 27 to 39 min in UTI89, in the Δ*cydAB* strain the doubling time increased from 37 to 106 min after treatment (Fig. 4F). Together, these data demonstrate that although cytochrome *bd* is dispensable for energy generation during planktonic growth, loss of cytochrome *bd* sensitizes bacteria to oxidative and nitrosative stress, consistent with previous studies on K-12 E. coli and the multidrug-resistant strain ST131 (35, 36).

While there was a trend toward increased doubling time in all strains after treatment with NOC-12, treatment of the Δ*cydAB* strain increased doubling time approximately 3-fold relative to its untreated control. This observation suggests that during aerobic growth cytochrome *bd* serves as an NO sink that reversibly sequesters NO and protects the more efficient cytochrome *bo*-mediated respiration. Accordingly, loss of cytochrome *bd* would decrease nitrosative stress resistance and render the dominant respiratory complex, cytochrome *bo*, susceptible to irreversible inhibition by NO. As such, treatment of the Δ*cydAB* strain with NO would poison all preformed cytochrome *bo* complexes in the membrane and force the bacteria to synthesize new oxidases prior to resuming growth. Consistent with this hypothesis, we observe a marked increase (∼10-fold relative to UTI89) of *cyoABCD* transcript in the interior of Δ*cydAB* colony biofilms, where NO is expected to be most abundant (Fig. 4H). These results contrast with previous studies in K-12 E. coli, in which loss of cytochrome *bd* induces a marked upregulation of *appBC* (26). These observations demonstrate that the regulation of quinol oxidases in UPEC is distinct from that previously defined in K-12 and suggest that cytochrome *bd* may serve as an NO sink in biofilms. In conjunction with the disrupted biofilm architecture and altered ECM abundance in Δ*cydAB* biofilms, these data suggest that cytochrome *bd*-expressing subpopulations are critical, not only for directing ECM biosynthesis but also for withstanding harmful metabolic by-products while in the biofilm state.

### Heterogeneous expression of quinol oxidases at the population level.

Our data thus far indicate that in addition to heterogeneity in quinol oxidase expression in the biofilm state, heterogeneous expression of quinol oxidases must also be occurring in the planktonic population. Our planktonic studies revealed a lag in growth of the Δ*cyoAB* and the Δ*cydAB* mutants when these strains were grown under ambient oxygen concentrations, suggesting that in a given culture there are subpopulations—like in the biofilm—that stochastically or deterministically express different respiratory components. Such a bet-hedging approach could provide UPEC with the flexibility to quickly adapt to a given niche, be it different locales in the genitourinary tract or in the gastrointestinal tract during host colonization. In the context of urinary tract infection, E. coli traverses from the nearly anoxic gut to the perineum, where it encounters atmospheric oxygen concentrations, prior to ascending the urethra to enter the hypoxic bladder, where the dissolved urinary oxygen concentration is 4 to 6% (40). This microbial journey is performed by planktonic cells, which can then expand into multicellular communities on and within bladder epithelial cells, as well as on urinary catheters (1, 41). In previous studies, we and others demonstrated that UPEC respire aerobically during infection (20–22) and that biofilm formation is favored under conditions that mimic oxygen levels in the bladder (19).

**FIG 5:**
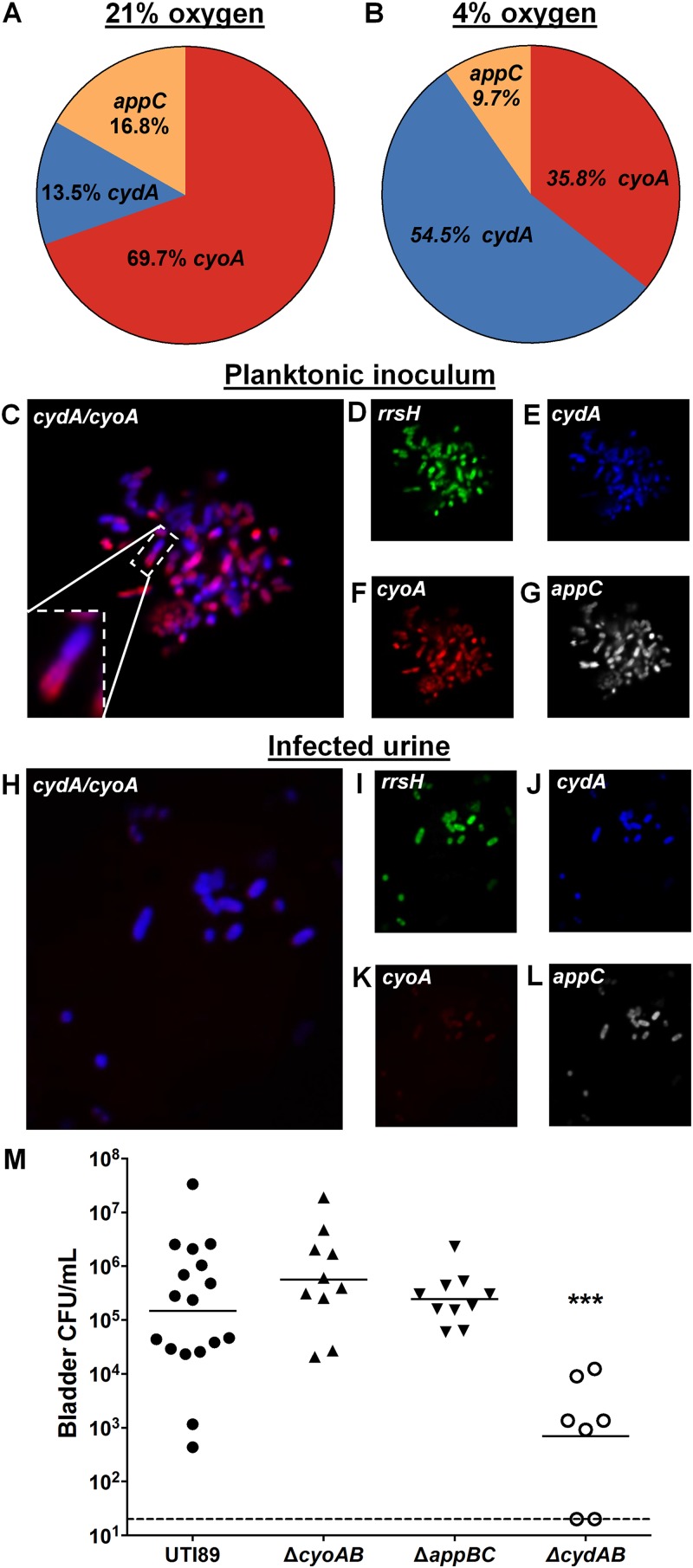
Respiratory heterogeneity provides a fitness advantage during urinary tract infection. (A and B) Pie charts depicting relative abundance of *cydA*, *cyoA*, and *appC* transcripts detected using RT-qPCR in planktonic cultures grown at 21% oxygen in the manner used to prepare cultures to inoculate mice (A), as well as planktonic cultures grown at 4% oxygen (B). Data are representative of three biological replicates. (C to G) PNA-FISH was used to detect quinol oxidase transcripts from cultures used to inoculate mice. Data are representative of three biological replicates. (H to L) PNA-FISH was used to detected quinol oxidase transcripts in the urine of mice infected with UTI89. Urine was pooled from 20 mice. (M) Graph depicting bladder titers obtained from mice infected with UTI89 or quinol oxidase mutant strains at 24 h postinfection. Each point represents a mouse. UTI89 and Δ*cydAB* strains are representative of two independent experiments. Δ*cyoAB* and Δ*appBC* strains are representative of one experiment. Statistical analysis was performed in GraphPad Prism using a two-tailed Mann-Whitney test. Line represents geometric mean. ***, *P* < 0.001.

The high abundance of *cydABX* transcript in the hypoxic areas of the biofilm, in conjunction with the defects observed in aerobically grown Δ*cydAB* planktonic cultures, raised the hypothesis that a cytochrome *bd*-expressing subpopulation exists in the planktonic state under ambient oxygen conditions and that this cytochrome *bd*-expressing subpopulation exhibits the greatest fitness advantage during infection. To test this hypothesis, we first analyzed transcript abundance in aerobic cultures used for inoculation during murine infections with RT-qPCR and PNA-FISH (Fig. 5). Under these conditions, the majority of transcript corresponds to *cyoABCD* (69.7%), with *cydABX* and *appBC* transcripts each comprising approximately 15% of detected transcripts (Fig. 5A). Transcript abundance was altered by decreasing ambient oxygen concentrations, with the most abundant transcript corresponding to *cydABX* in 12%, 8%, and 4% oxygen, the last being the concentration of dissolved oxygen concentration in the urine (Fig. 5A and B and Fig. S8) (40). This shift in transcript abundance is largely due to a marked induction of *cydABX* expression under hypoxic conditions (Fig. S9). PNA-FISH analysis revealed the presence of bacteria which uniquely express cytochrome *bo* (Fig. 5F), *bd* (Fig. 5E), or *bd*_2_ (Fig. 5G), as well as some cells that have transcript of all three operons (Fig. 5C and E to G). Intriguingly, we observed dividing cells in which each daughter had distinct quinol oxidase transcript abundance (Fig. 5C, inset), suggesting that asymmetric distribution of respiratory transcripts during division may be a mechanism by which these subpopulations are generated. This hypothesis is supported by previous studies in E. coli demonstrating that quinol oxidases exhibit unusually noisy gene expression and that asymmetric cell division is a major generator of heterogeneity (42–44).

10.1128/mBio.02400-18.8 FIG S8 Ambient oxygen concentration influences quinol oxidase transcript abundance. Pie charts depicting relative abundance of *cydA*, *cyoA*, and *appC* transcripts detected using RT-qPCR in planktonic cultures grown at 12% (A) or 8% (B) oxygen. Data are representative of three biological replicates. Download FIG S8, TIF file, 2.0 MB. Copyright © 2019 Beebout et al. 2019 Beebout et al. This content is distributed under the terms of the Creative Commons Attribution 4.0 International license.

10.1128/mBio.02400-18.9 FIG S9 Expression of quinol oxidases as a function of oxygen tension. (A) RT-qPCR data depicting relative fold difference in abundance of each quinol oxidase transcript of planktonic bacteria grown at 4% oxygen compared to 21% oxygen. (B) Relative abundance of each transcript at 21% and 4% oxygen compared to *gyrB*. (C) Raw *C_T_* values for each transcript at 21% and 4% oxygen. Data are presented as mean ± SEM. All data are representative of three biological replicates. Download FIG S9, TIF file, 0.9 MB. Copyright © 2019 Beebout et al. 2019 Beebout et al. This content is distributed under the terms of the Creative Commons Attribution 4.0 International license.

### Expression of cytochrome *bd* is dominant during acute urinary tract infection.

Previous studies reported that deletion of cytochrome *bd* impairs UPEC virulence in a UTI model (36). To gauge the contribution of each quinol oxidase during infection, we evaluated the fitness of Δ*cyoAB*, Δ*appBC*, and Δ*cydAB* mutants compared to the parent strain in a murine model of acute urinary tract infection. Consistent with the previous report (36), the Δ*cydAB* mutant exhibited an ∼2-log decrease in bladder colonization by 24 h relative to the parent strain, while the mutants deleted for *cyoAB* and *appBC* colonized mice at the same level as the parent strain (Fig. 5M). Subsequent PNA-FISH on pooled urine obtained from mice infected with the parent strain revealed a marked enrichment in cytochrome *bd*-expressing cells and a corresponding reduction in the number of cells expressing cytochrome *bo* (Fig. 5H to L). This suggests either that the bladder environment induces transcription of *cydABX* or that only subpopulations of bacteria expressing *cydABX* are capable of efficiently colonizing the bladder. Together these data reveal the presence of subpopulations of bacteria that differentially express quinol oxidases as a potential bet-hedging mechanism to promote bladder colonization.

## DISCUSSION

Cytochrome *bd* is a multifunctional protein that is central to respiration and can maintain activity in the face of nitrosative stress (34). As such, bacteria expressing cytochrome *bd* presumably exhibit a fitness advantage under growth conditions that are low in oxygen or high in metabolic by-products that increase nitric oxide concentration. The biofilm state, while protecting the bacterial residents from predation and desiccation, constitutes a high-density environment with several chemical gradients that result from the consumption and production of metabolites. Accordingly, expressing an enzyme that can facilitate tolerance to metabolic by-products, such as nitric oxide, would ensure that biofilm residents do not perish as a consequence of their own metabolic excretions. Our study elucidates the distribution of quinol oxidase expression in the biofilm state and indicates that the bulk of biofilm residents express cytochrome *bd*, particularly in the densely populated interior. The cytochrome *bd*-expressing bacteria are not necessarily using cytochrome *bd* for respiration, as many of them also have low levels of cytochrome *bo* and *bd*_2_ transcripts (Fig. 2 and Fig. S3 and S4). Rather, the production of cytochrome *bd* may be leveraged toward providing tolerance to nitrosative stress, which irreversibly inhibits cytochrome *bo*. Indeed, in Δ*cydAB* biofilms we observe a marked increase in cytochrome *bo* expression (Fig. 4H), suggesting that loss of cytochrome *bd* impairs nitric oxide tolerance and that increased production of cytochrome *bo* may be a compensatory mechanism that allows biofilm bacteria to respire in the presence of high levels of nitric oxide.

In addition to acting as a respiratory inhibitor, nitric oxide regulates cyclic di-GMP abundance and thereby governs the switch from motility to aggregation and biofilm expansion (45, 46). Consequently, if cytochrome *bd* decreases nitric oxide availability, it would indirectly influence ECM production. Consistent with this hypothesis, loss of the cytochrome *bd*-expressing subpopulation reduces the total abundance of matrix components and leads to gross alterations of biofilm architecture (Fig. 3). It is thus possible that the cytochrome *bd-*expressing subpopulation is critical for promoting the biosynthesis of the ECM by influencing the nitric oxide–cyclic di-GMP signaling axis. We are currently investigating this possibility.

Most importantly, this work revealed the presence of planktonic subpopulations that express distinct quinol oxidases during growth. In conjunction with the observation that only cytochrome *bd* expression is critical for fitness during infection, this finding suggests that basal expression of cytochrome *bd* under aerobic conditions serves as a bet-hedging mechanism that promotes the expansion of bacteria during the transition from the aerobic perineum to the hypoxic bladder. In addition to allowing for efficient respiration in the hypoxic bladder, expression of cytochrome *bd* provides resistance against nitrosative stress—a metabolic by-product and component of the innate immune response—and promotes the formation of resilient biofilm communities. Alternatively, cytochrome *bd* may serve as an oxygen scavenger, thereby reducing oxygen tension and allowing distinct UPEC subpopulations to utilize anaerobic respiratory pathways. Consistent with this hypothesis, the alternative terminal electron acceptors nitrate and trimethylamine oxide (TMAO) are known to be present in the urine, and the anaerobic reduction of nitrate to nitrite by *Enterobacteriaceae* is the basis of a commonly used clinical test used to diagnose urinary tract infection. Together our observations suggest the presence of respiratory bet-hedging behavior in UPEC and additionally suggest the possibility of targeting heterogeneity as a method for homogenizing bacterial populations and impeding their ability to colonize the urinary tract.

## MATERIALS AND METHODS

### Bacterial strains.

All studies were performed in Escherichia coli cystitis isolate UTI89 (47). All gene deletions (Δ*cyoAB*, Δ*appBC*, and Δ*cydAB*) were performed using the λ-red recombinase system (48). Complementation constructs were created in plasmid pTRC99a with *cydABX* under the control of its native promoter as previously described (49). Primers used for gene deletions and complementation plasmid construction are listed in Table S1 in the supplemental material.

### Growth conditions.

For all analyses, strains were propagated overnight at 37°C with shaking in lysogeny broth (LB) (Fisher) at pH 7.4. To form colony biofilms, 10 µl of overnight culture was spotted onto 1.2× yeast extract-Casamino Acids (YESCA) agar (8) and allowed to grow at room temperature. Growth curves to assess tolerance to nitrosative or oxidative stress were performed in LB broth at 37°C with shaking, starting from an overnight culture normalized to optical density at 600 nm (OD_600_) of 0.05. At 2 h post-inoculation, cultures were split into equal volumes and treated with 0.5 mM NOC-12, 1 mM H_2_O_2_ or left unperturbed. OD_600_ and CFU-per-ml measurements were taken every hour for 8 h.

### RT-qPCR.

RNA was extracted from day 11 colony biofilms or planktonic cultures using the RNeasy kit (Qiagen). RNA was DNase treated using Turbo DNase I (Invitrogen) and reverse transcribed using SuperScript III reverse transcriptase (Invitrogen). cDNA was amplified in an Applied Biosystems StepOne Plus Real-Time PCR machine using TaqMan MGB chemistry with the primers and probes listed in Table S1. All reactions were performed in triplicate with four different cDNA concentrations (100, 50, 25, or 12.5 ng per reaction). Relative fold difference in transcript abundance was determined using the ΔΔ*C_T_* method (50) with target transcripts normalized to *gyrB* abundance from a total of 3 to 4 biological replicates.

### Peptide nucleic acid fluorescence *in situ* hybridization (PNA-FISH).

Day 11 biofilms were flash frozen in Tissue-Tek O.C.T. compound (Electron Microscopy Sciences) and cryosectioned as described previously (4). The PNA-FISH hybridization protocol was adapted from the work of Almeida et al. (51). Biofilm cryosections were fixed in 4% paraformaldehyde (PFA) for 30 min at room temperature and then dehydrated for 10 min in 50% ethanol. After dehydration, 100 μl of hybridization solution (see below for details) was applied to the slides. All hybridizations were performed at 60°C for 30 min. Next, slides were submerged in prewarmed wash solution for 30 min, mounted using ProLong Diamond (ThermoFisher), and imaged using a Zeiss 710 confocal laser scanning microscope (CLSM). For planktonic cells, 1 ml of culture was sedimented, fixed in 4% PFA, resuspended in 50% ethanol, incubated at −20°C for 30 min, and resuspended in 100 μl hybridization solution. After hybridization, cells were pelleted, resuspended in 500 μl prewarmed wash solution, and incubated at 60°C for 30 min. Finally, cells were pelleted and resuspended in 100 μl sterile water before being applied to microscope slides for imaging. Wash solution contained 5 mM Tris-HCl (pH 7.4), 15 mM NaCl, and 1% Triton X-100. Hybridization solution contained 10% (wt/vol) dextran sulfate, 30% formamide, 50 mM Tris-HCl (pH 7.4), 10 mM NaCl, 5 mM EDTA, 0.1% Triton X-100, and 200 nM (each) PNA-FISH probe. Probe sequences were based on the probes used for qPCR (efficiency of hybridization, *rrsH*, 81; *cydA*, 107; *cyoA*, 115; *appC*, 73) and were synthesized by PNA Bio (Newbury Park, CA).

### ATP measurements.

ATP was quantified from mid-log (4 h after subculture) planktonic cultures using the Cell-Glo Titer kit (Promega). Cultures were normalized to an OD_600_ of 0.5, pelleted, and resuspended in PBS. Fifty microliters of bacterial suspension was mixed with an equal volume of Cell-Glo Titer reagent and incubated with shaking at room temperature for 15 min. After incubation, luminescence was measured on a SpectraMax i3 plate reader (Molecular Devices). Luminescence was converted to concentration of ATP using a standard curve on the same plate.

### Extracellular matrix extraction.

Extracellular matrix was extracted using established methods (28). Briefly, biofilms were grown on YESCA agar containing 25 µg/ml Congo red. After 60 h, biofilms were homogenized in cold 10 mM Tris-HCl (pH 7.4) using an Omni Tissue Homogenizer (motor speed 9) five times for 1 min per cycle. Next, the homogenate was centrifuged three times for 10 min at 5,000 × *g* to remove cells. The supernatant was spiked with NaCl (final concentration, 170 mM) and centrifuged for 1 h at 13,000 × *g* to pellet the matrix. The ECM pellet was washed in 10 mM Tris-HCl, pH 7.4, with 4% SDS and incubated at room temperature with rocking overnight. Next, the suspended ECM was centrifuged at 13,000 × *g* for 1 h, resuspended in cold 10 mM Tris-HCl (pH 7.4), and centrifuged at 30,000 × *g* for 20 min. Pelleted ECM was resuspended in MQ water and flash frozen.

### Congo red depletion assays.

ECM abundance was quantified using Congo red depletion assays adapted from established protocols (52). Colony biofilms grown on YESCA agar were harvested into PBS at specific time points and homogenized. Congo red (40 µg/ml, final concentration) was added to homogenized biofilms, which were then incubated at 37°C for 1 h. After incubation, ECM was pelleted by centrifugation, the supernatant was removed, and supernatant absorbance (490 nm) was measured using a SpectraMax i3 plate reader (Molecular Devices).

### Solid-state NMR measurements.

All NMR experiments were performed in an 89-mm-bore 11.7T magnet using either an HCN Agilent probe with a DD2 console (Agilent Technologies) or a home-built four-frequency transmission line probe with a Varian console. Samples were spun at 7,143 Hz in either 36-µl-capacity 3.2-mm zirconia rotors or thin-walled 5-mm-outer-diameter zirconia rotors. The temperature was maintained at 5°C with an FTS chiller (FTS Thermal Products, SP Scientific, Warminster, PA) supplying nitrogen at −10°C. The field strength for ^13^C cross-polarization was 50 kHz with a 10% ^1^H linear ramp centered at 57 kHz. The cross-polarization magic angle spinning (CPMAS) recycle time was 2 s for all experiments. ^1^H decoupling was performed with continuous wave decoupling. ^13^C chemical shifts were referenced to tetramethylsilane as 0 ppm using a solid adamantine sample at 38.5 ppm. The 15.6-mg wild-type ^13^C CPMAS spectrum was the result of 32,768 scans, and the 8.3-mg mutant spectrum was the result of 100,000 scans. NMR spectra were processed with 80-Hz line broadening.

### SDS-PAGE gels.

A portion of the lyophilized ECM sample used for solid-state NMR analysis was resuspended in 98% formic acid and vacuum centrifuged. The samples were then reconstituted in SDS-PAGE sample buffer containing 8 M urea and 50 mM DTT and further diluted to desired concentrations. All samples were centrifuged briefly at 10,000 × *g* to remove any insoluble material and used for electrophoresis. The gels were stained with instant blue and destained in water.

### Immunofluorescence.

An immunofluorescence assay targeting CsgA, the major curli subunit, was performed as previously described (9). Biofilm cryosections were fixed in 4% PFA for 30 min at room temperature and blocked overnight in 5% BSA at 4°C. Sections were washed in PBS, incubated with rabbit anti-CsgA antibodies (GenScript) (1:1,000) at room temperature for 1 h, washed in PBS, and incubated with Alexa Fluor 647 goat anti-rabbit IgG (ThermoFisher) (1:1,000) at room temperature for 1 h. Slides were counterstained with SYTO 9 and imaged using confocal laser scanning microscopy (CLSM).

### Murine infections.

Murine infections were performed as described previously (53). In brief, UTI89 and each mutant strain were inoculated individually into 5 ml LB medium and grown with shaking at 37°C for 4 h. Next, this culture was diluted 1:1,000 into 10 ml fresh medium and grown statically at 37°C for 24 h. After 24 h, this culture was diluted 1:1,000 into 10 ml fresh medium and grown for another 24 h at 37°C statically. Next, 7- to 8-week-old C3H/HeN female mice were transurethrally inoculated with 50 µl PBS containing 10^7^ CFU bacteria. Mice were sacrificed at 24 h postinfection, after which bladders were removed and homogenized for CFU enumeration. All animal studies were approved by the Vanderbilt University Medical Center Institutional Animal Care and Use Committee (IACUC) (protocol numbers M/12/191 and M1500017-01) and carried out in accordance with all recommendations in the *Guide for the Care and Use of Laboratory Animals* (54) of the National Institutes of Health and the IACUC.

### Statistical analysis.

All statistical analyses were performed in GraphPad Prism using the most appropriate test. Details of test used, error bars, and statistical significance cutoffs are presented in the figure legends.
