# Supplementary figures and images for: Respiratory Heterogeneity Shapes Biofilm Formation and Host Colonization in Uropathogenic Escherichia coli
Source: mBio. 2019 Apr 2;10(2):e02400-18. doi: 10.1128/mBio.02400-18 (PMC6445943; doi:10.1128/mBio.02400-18)

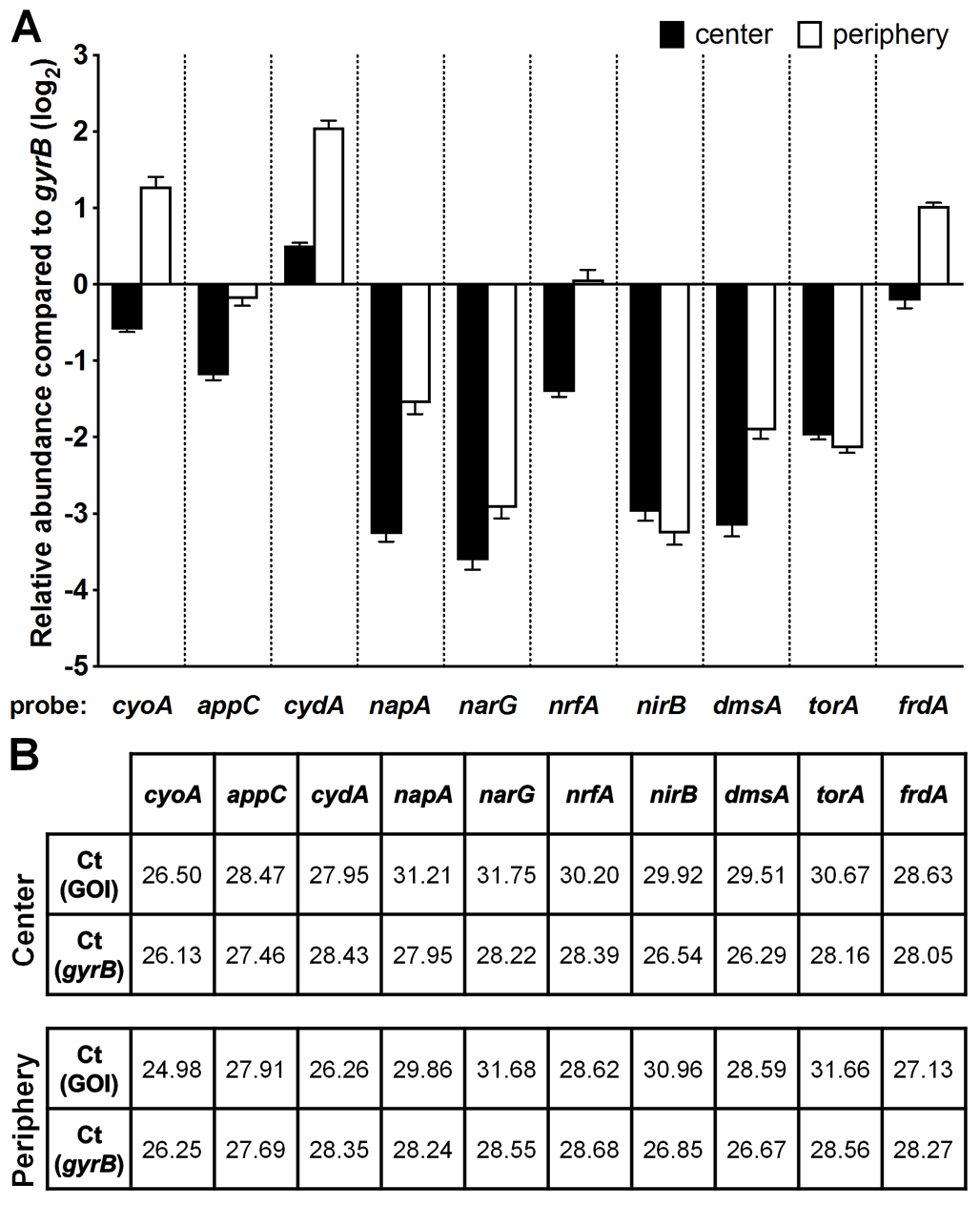

Supplement: FIG S1 [file mBio.02400-18-sf001.tif]

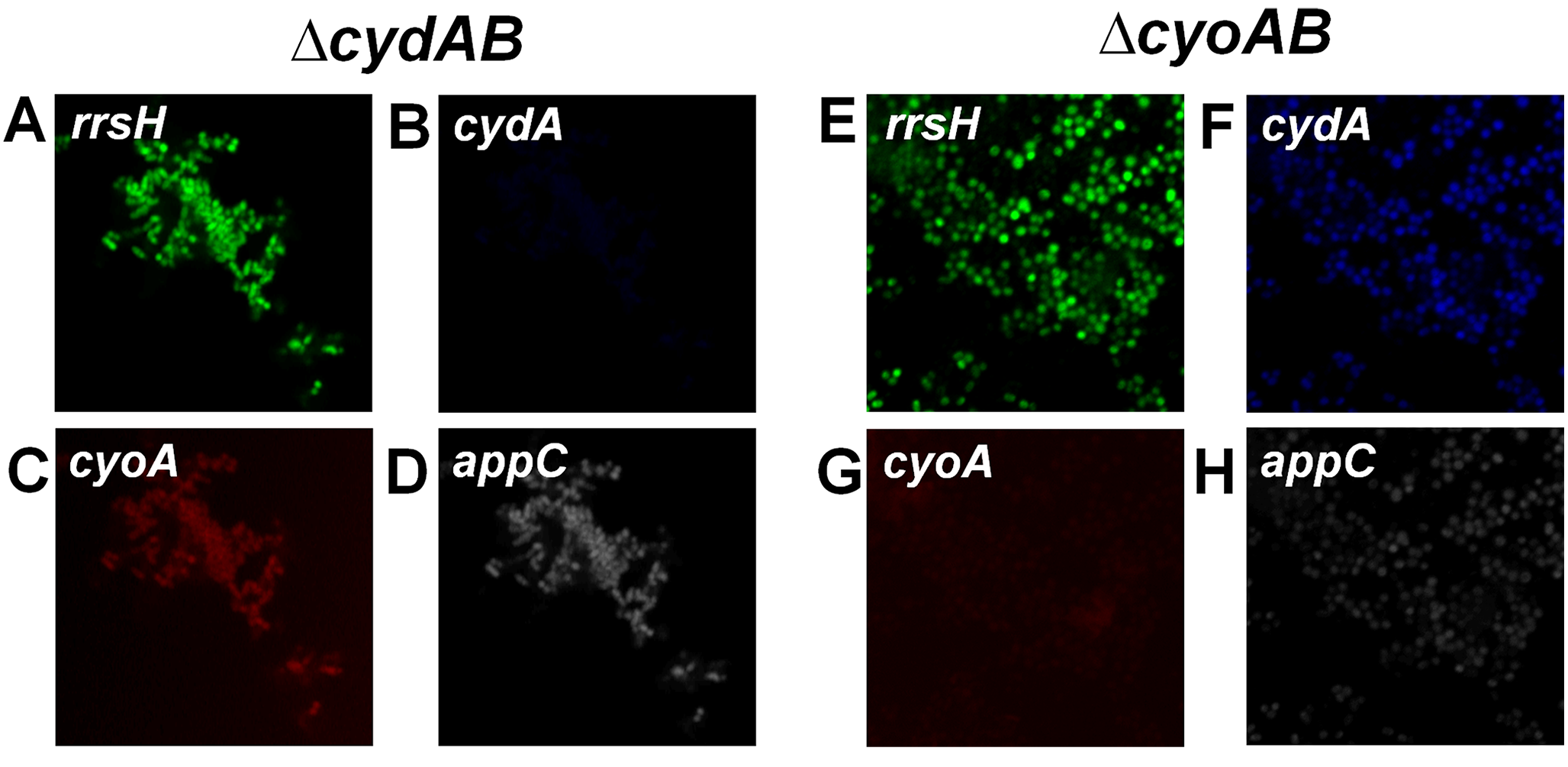

Supplement: FIG S2 [file mBio.02400-18-sf002.tif]

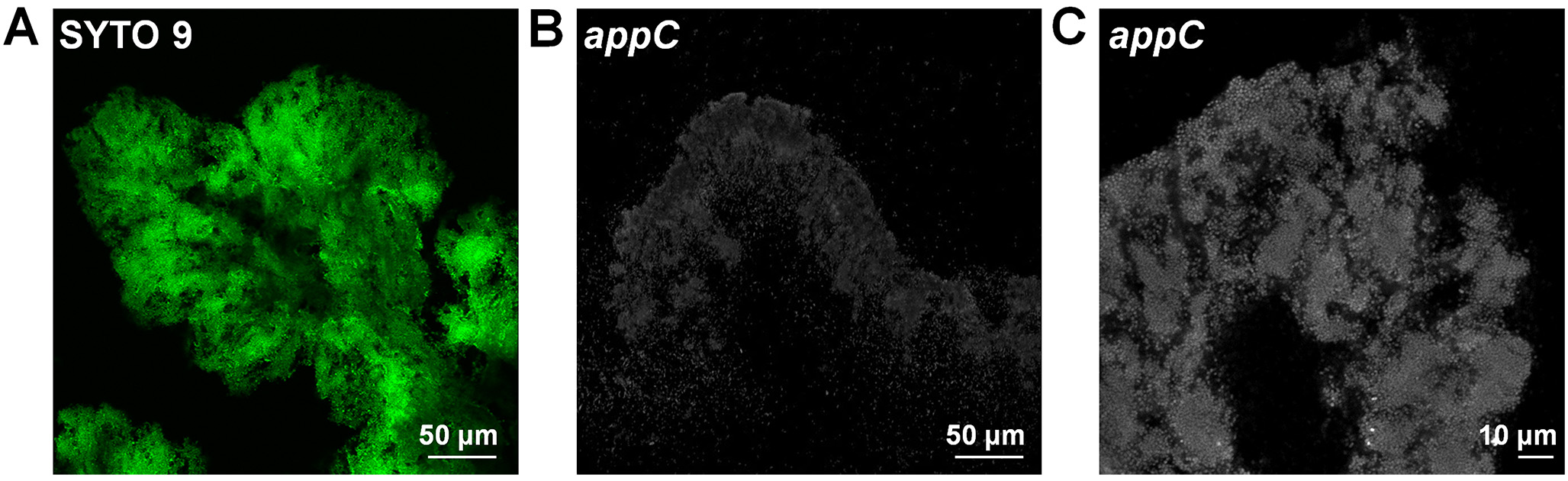

Supplement: FIG S3 [file mBio.02400-18-sf003.jpg]

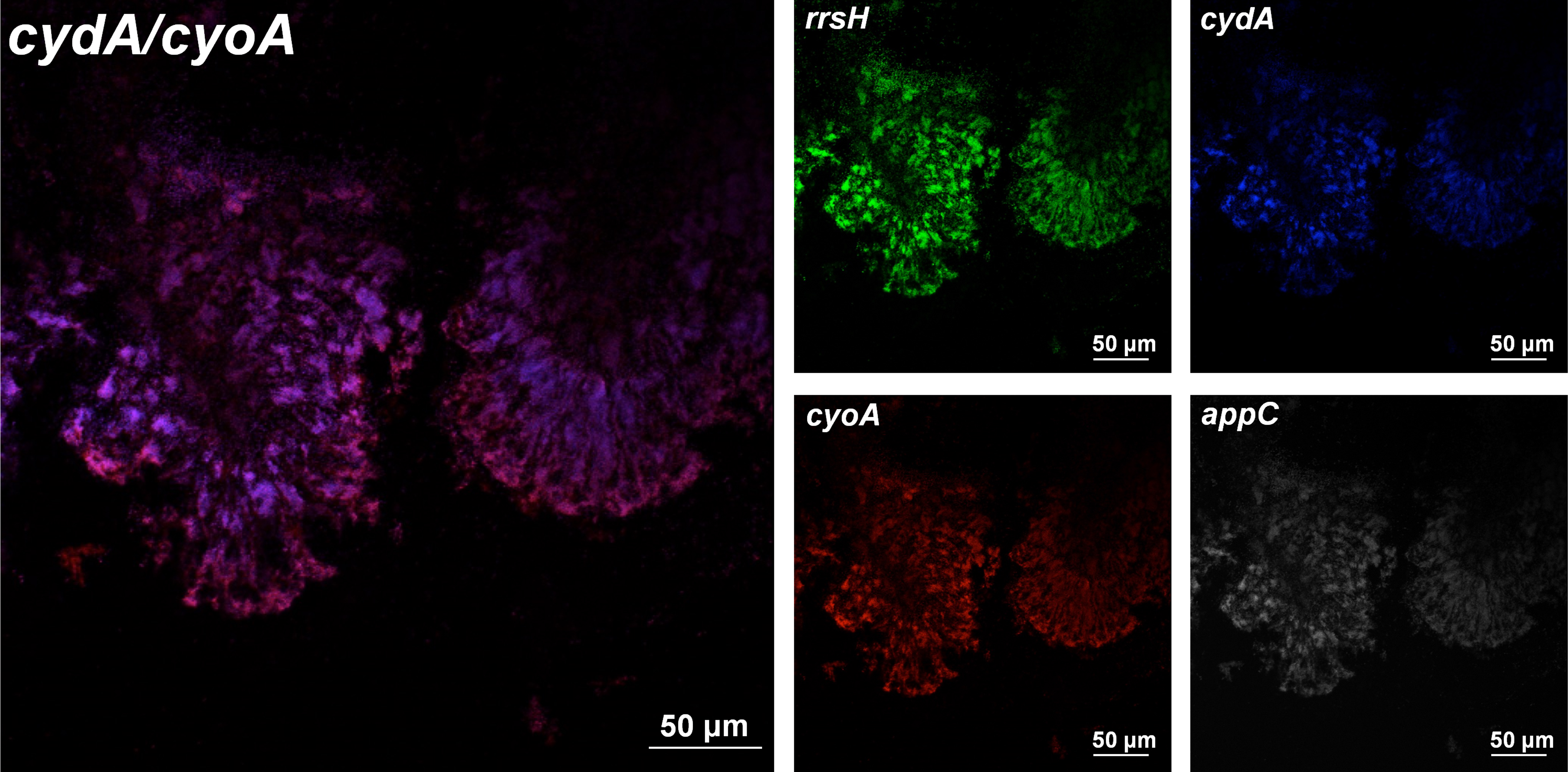

Supplement: FIG S4 [file mBio.02400-18-sf004.jpg]

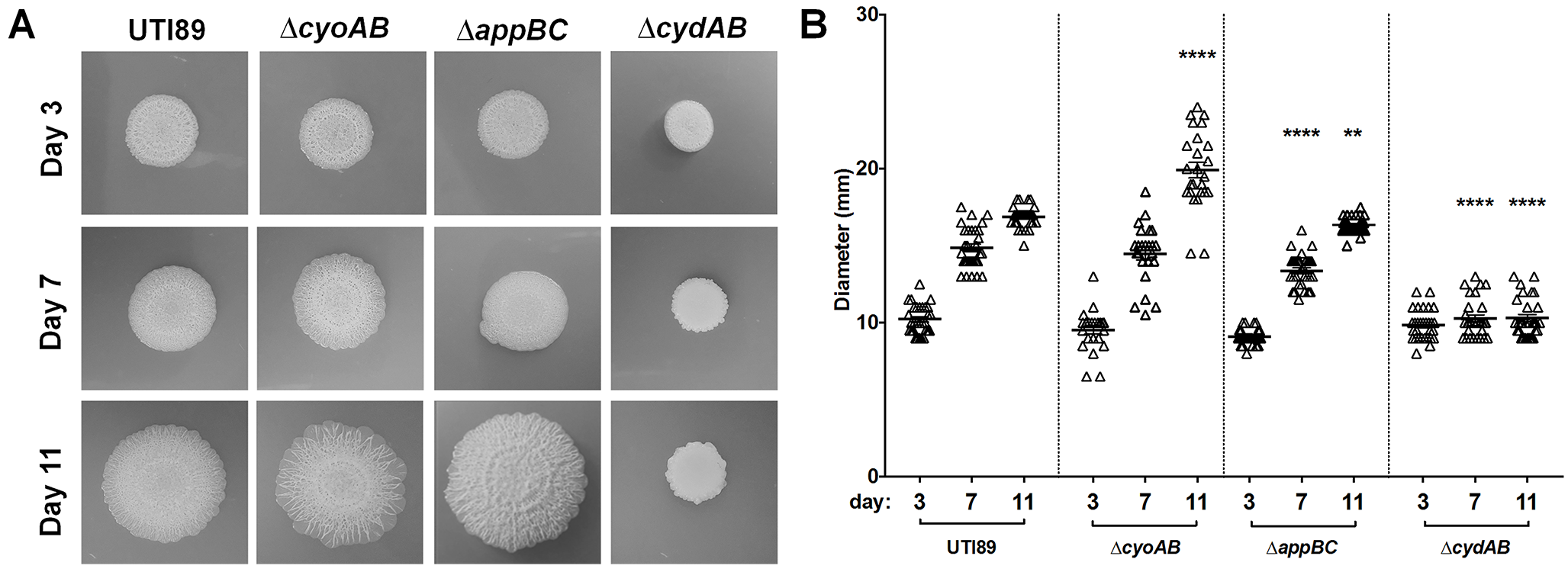

Supplement: FIG S5 [file mBio.02400-18-sf005.tif]

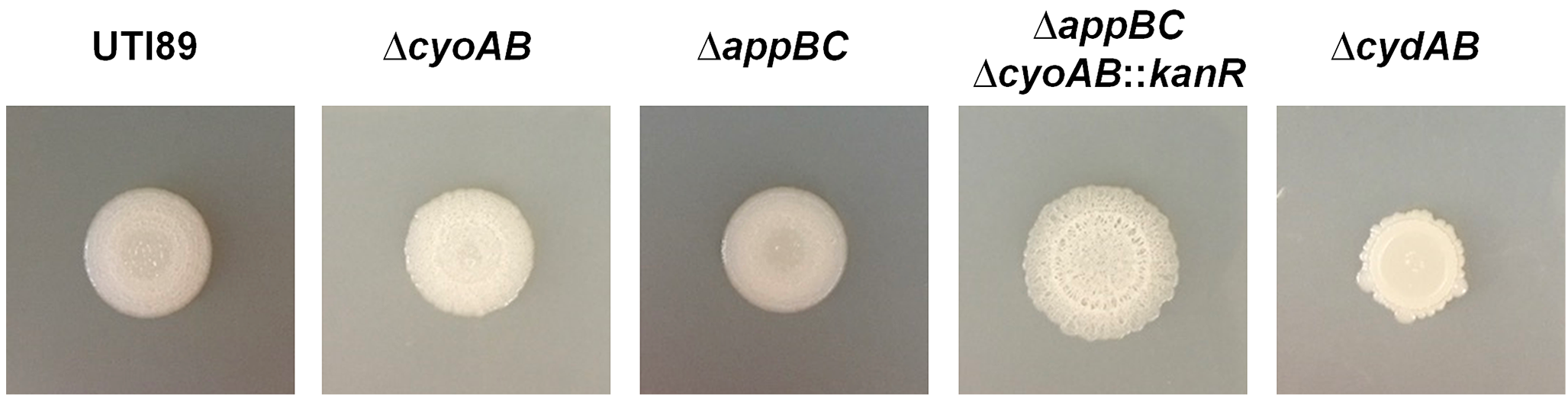

Supplement: FIG S6 [file mBio.02400-18-sf006.tif]

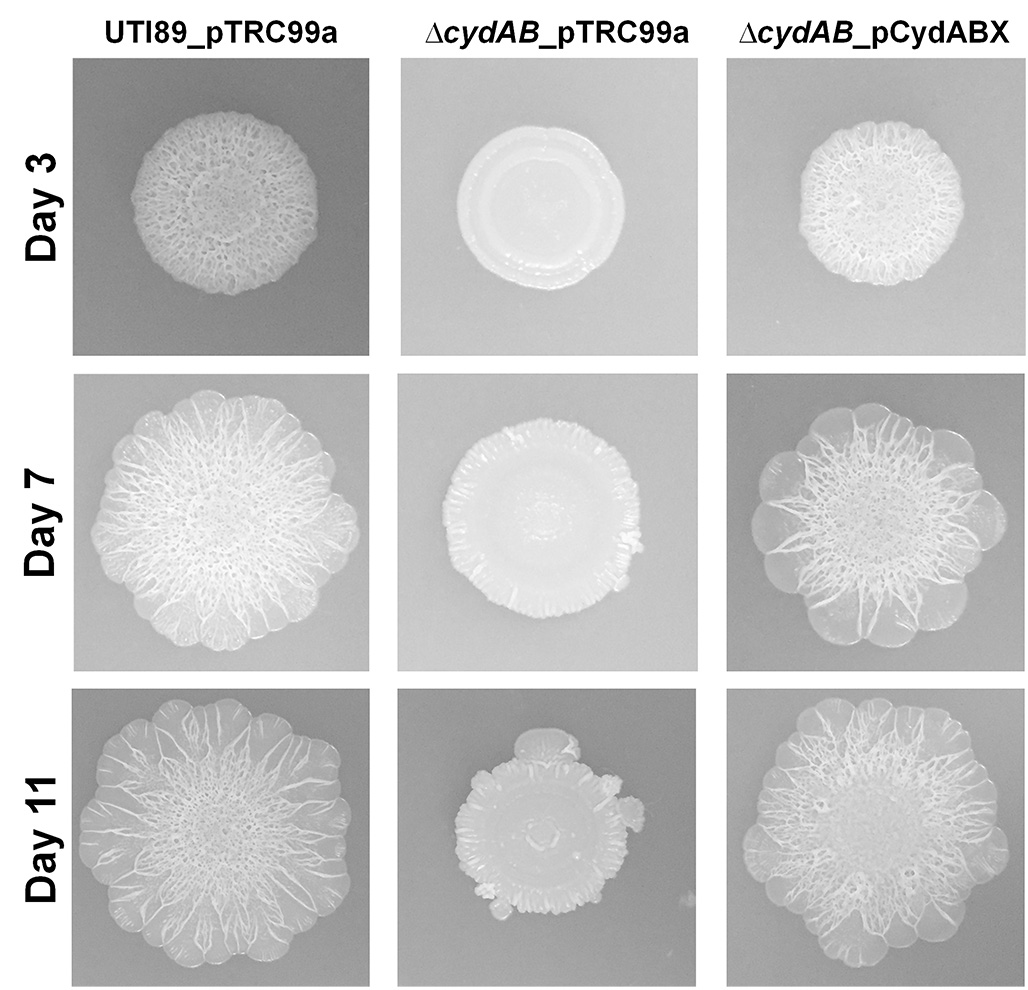

Supplement: FIG S7 [file mBio.02400-18-sf007.jpg]

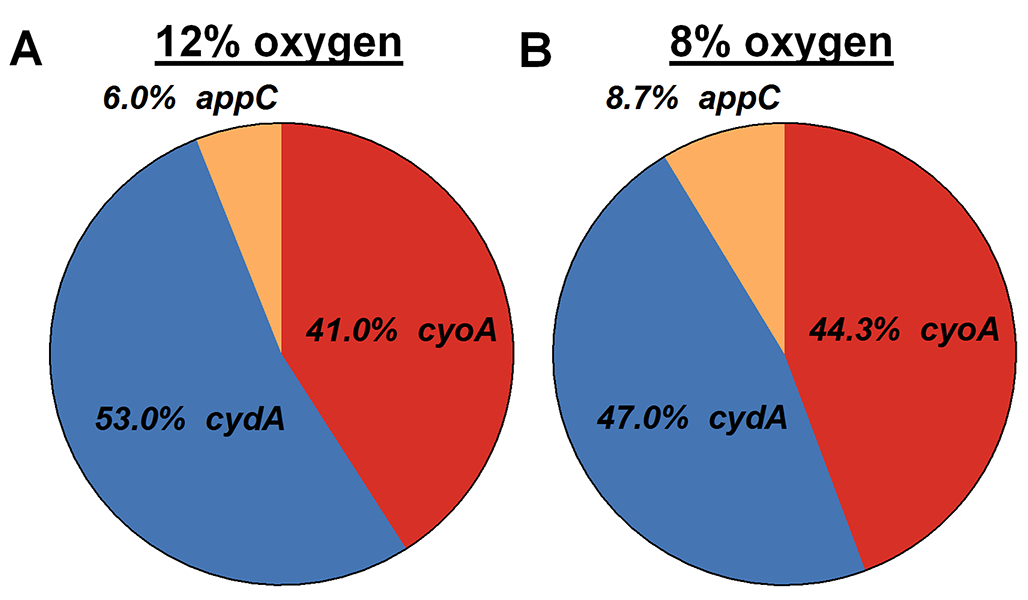

Supplement: FIG S8 [file mBio.02400-18-sf008.tif]

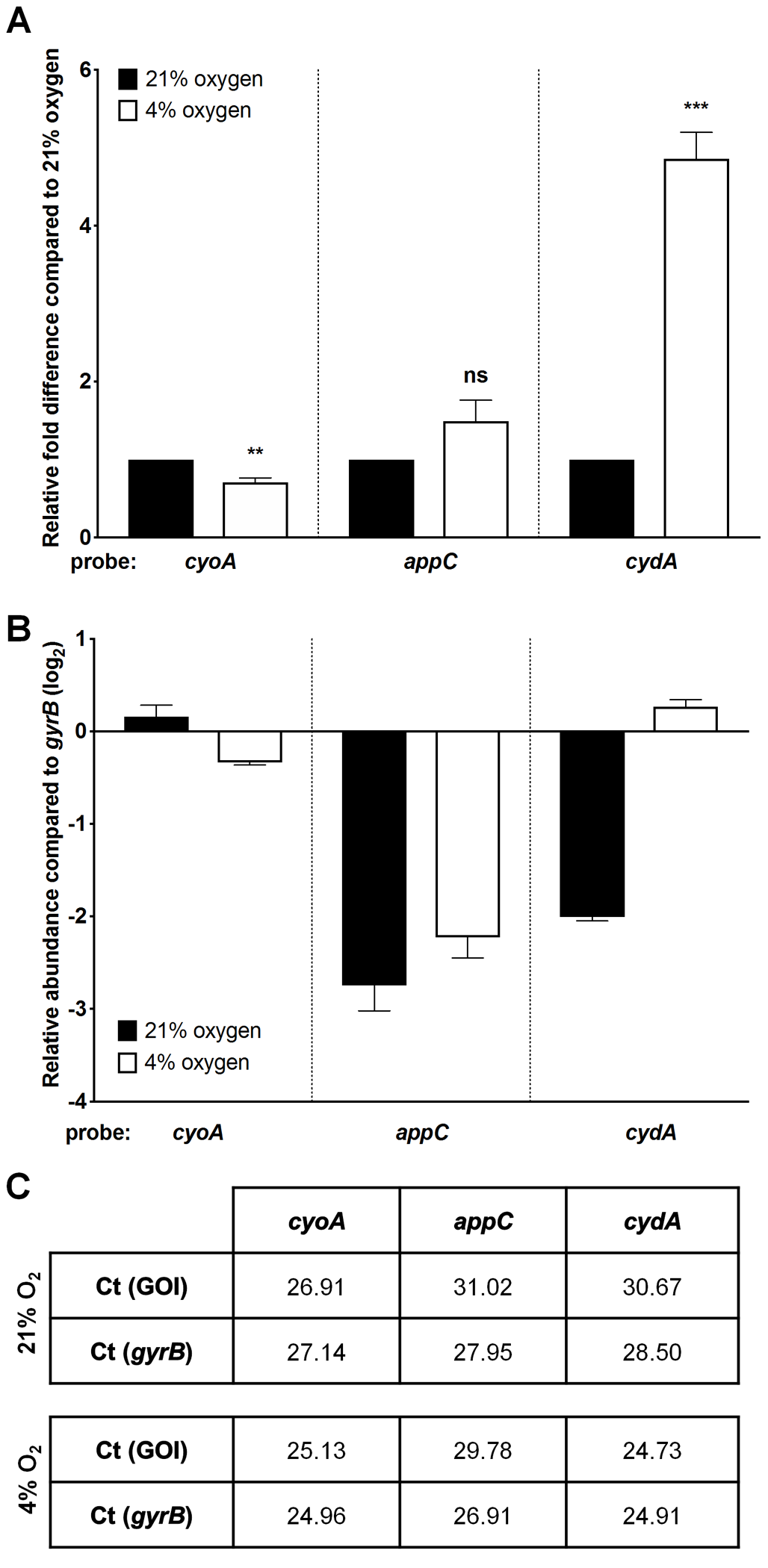

Supplement: FIG S9 [file mBio.02400-18-sf009.tif]
