# Supplementary material for: Respiratory Heterogeneity Shapes Biofilm Formation and Host Colonization in Uropathogenic Escherichia coli
Source: mBio. 2019 Apr 2;10(2):e02400-18. doi: 10.1128/mBio.02400-18 (PMC6445943; doi:10.1128/mBio.02400-18)
Supplement: TABLE S1 [file mBio.02400-18-st001.docx]

**Table S1: Primers and probes**

| **Primer/Probe** | **Sequence (5’ 🡪 3’)** | **Purpose** |
| --- | --- | --- |
| cydA_KO_Fwd | ATGATGTTAGATATAGTCGAACTGTCGCGCTTACAGTTTGCCTTGACCGCGATGTACGTGTAGGCTGGAGCTGCTTC | *cydAB* knockout |
| cydB_KO_Rev | GTTACGTTCAATATCTTCTTTGGTGATACGACCGAACATTTTCCAGTCATATGAATATCCTCCTTAG | *cydAB* knockout |
| cydA_KO_Test_Fwd | GATCAAATTGGTGAGATCGTGAC | *cydAB* knockout |
| cydB_KO_Test_Rev | CTAACAGAAGTGCCATCACG | *cydAB* knockout |
| cydA1_Fwd_XbaI | CTGCAGTCTAGACTGGTCAAGTTATCCATCATTCACT | *cydABX* complementation |
| cydX_Rev_SacI | CGTATTGAGCTC/TTGCGATAATCTTACTCATCAGATGTC | *cydABX* complementation |
| appC_KO_Fwd | ATGTGGGATGTCATTGATTTATCGCGCTGGCAGTTTGCTCTGACCGCGGTGTAGGCTGGAGCTGCTTC | *appBC* knockout |
| appB_KO_Rev | TTAGTACAGCTCGTTTTCGTTACGGCGGAGAGTTTCTGTCGTCATGCCATATGAATATCCTCCTTAG | *appBC* knockout |
| appC_KO_Test_Fwd | ACGCAGACGTCACGGCG | *appBC* knockout |
| appB_KO_Test_Rev | TGCACAGTCAGGTGCCAGC | *appBC* knockout |
| cyoB_KO_Fwd | TCAGTTGCCATTTTTCAGCCCTGCCTTAGTAATCTCATCGGTGTAGGCTGGAGCTGCTTC | *cyoAB* knockout |
| cyoA_KO_Rev | GTCATTATTTGCAGGCACTGTATTGCTCAGTGGCTGTAATTCTGCGCTGCATATGAATATCCTCCTTAG | *cyoAB* knockout |
| cyoB_KO_Test_Fwd | CATCCAGATAAGACCGGAAGTG | *cyoAB* knockout |
| cyoA_KO_Test_Rev | GCAACATATGTGACCTGATAGC | *cyoAB* knockout |
| cyoA_Fwd | CAATGCCCTGTTCCGGGTAGATG | qPCR |
| cyoA_Rev | ACGGTACCTATCTTAATCATCATCTTCC | qPCR |
| cyoA probe | NED-TCGTCGTGTGCCAGCGGCTTG | qPCR |
| appC_Fwd | CGATGTCGCATTACGCTCG | qPCR |
| appC_Rev | GGTGCAGGTTCTGTTTGCCACT | qPCR |
| appC probe | NED-CTGCGTATGAAGTCGCGCAAG | qPCR |
| cydA_Fwd | GTGGCTACCGGTCTGACCATG | qPCR |
| cydA_Rev | CCAACCGAAGAAGAACAGACCTAC | qPCR |
| cydA probe | FAM-CGCTGGCAATCGAAGGTCTGATG | qPCR |
| narG_Fwd | TCTCGCTATACTGGACACCTGA | qPCR |
| narG_Rev | CCGTATAGTCCACCGGATTCAT | qPCR |
| narG probe | NED-TGCCGTCTGCGCCGCAGT | qPCR |
| napA_Fwd | CTTCCGCGTGTGGTACTGC | qPCR |
| napA_Rev | GTGCCGCTCGGGATATTCC | qPCR |
| napA probe | FAM-CGTCTGCCTGCGGACATGGTGGTGAC | qPCR |
| nrfA_Fwd | GGTCAGTGCCATGTGGAGT | qPCR |
| nrfA_Rev | CGACAGGGAGTTAGTCCAGTCA | qPCR |
| nrfA probe | NED-CCGTGGGATGACGGCATGAAAGTCGAA | qPCR |
| nirB_Fwd | CTGGTGCTGAACGCTATCG | qPCR |
| nirB_Rev | ATAGCAGCAATCAGGTCGC | qPCR |
| nirB probe | FAM-AACTGCCGGACAGCGCGCAAATCTG | qPCR |
| frdA_Fwd | CCTCGACCTGCGTCACCTCGGC | qPCR |
| frdA_Rev | TTCTGGCACGAATGGCGTA | qPCR |
| frdA probe | FAM-CGGATCGACGCCAACGTA | qPCR |
| dmsA_Fwd | GCT ATC TCG ATG CTG GCG A | qPCR |
| dmsA_Rev | GTTCAATGGCATCGGTCCAC | qPCR |
| dmsA probe | NED-CGGCGCGCGCGAAGGTTCATACAGCTTAC | qPCR |
| torA_Fwd | CGTGGATGATTGTCGTTCTGG | qPCR |
| torA_Rev | TTGTCGTGAACAGGCGGA | qPCR |
| torA probe | NED-GGCTGGCACTATAACGGCGCAGGCAC | qPCR |
| gyrB_Fwd | GATGCGCGTGAAGGCCTGATTG | qPCR |
| gyrB_Rev | CACGGGCACGGGCAGCATC | qPCR |
| gyrB probe | VIC-ACGAACTGCTGGCGGA | qPCR |
| cyoA PNA | CGTCGTGTGCCA – Lys (Atto425) | PNA-FISH |
| cydA PNA | CGATTGCCAGCG – Lys (Cy5) | PNA-FISH |
| appC PNA | TGCGCGACTTCATA – Lys (TexasRed) | PNA-FISH |
| rrsH PNA | AGTAATTCCGATTAACG – Lys (Atto532) | PNA-FISH |
